# Does heightened subjective sexual arousal lower pain in women if disgust is minimized?

**DOI:** 10.1371/journal.pone.0323095

**Published:** 2025-05-20

**Authors:** Lara Lakhsassi, Charmaine Borg, Peer Briken, Peter J. de Jong

**Affiliations:** 1 Department of Clinical Psychology and Experimental Psychopathology, University of Groningen, Groningen, The Netherlands; 2 Institute for Sex Research, Sexual Medicine, and Forensic Psychiatry, University Medical Center Hamburg-Eppendorf, Hamburg, Germany.; PLoS ONE, UNITED STATES OF AMERICA

## Abstract

It has been proposed that acute pain can generally be reduced by sexual activity. Increasing subjective sexual arousal might thus help reduce pain during sex. Yet, in conflict with the view that subjective sexual arousal would generally reduce pain, previous research has failed to find that presenting a sexually arousing film stimulus attenuates pain during a cold pressor test (CPT) in women. This might be due to the sexually arousing film having also elicited disgust. Therefore, this study tested whether subjective sexual arousal could generally reduce pain, provided that concurrently-elicited disgust is minimized. Female undergraduates with no sexual dysfunction were randomly distributed through a digital list randomizer to either watch: a porn film that can elicit similar levels of disgust and sexual arousal, a porn film that elicits greater subjective sexual arousal than disgust, or a neutral train ride film (*N* = 174). A CPT was utilized for pain induction while simultaneously viewing the assigned films. Pain was indexed by subjective ratings of pain intensity, and CPT duration. The results showed no differences in pain intensity or pain tolerance across conditions. Thus, in this study, disgust appeared no critical moderator of the relationship between sexual arousal and pain. The findings converge to the conclusion that, in women, subjective sexual arousal does not generally reduce the experience of pain. This might also imply that increasing subjective sexual arousal alone might not be sufficient to reduce genital pain, though further research is needed to test this inference given that pain sensitivities may differ across the body.

## Introduction

In healthy, functional sexual interactions, people generally develop several positive associations with sex. Such associations usually follow previous rewarding and pleasurable sexual experiences, and contribute to the future pursuit of sexual interactions [1]. However, sexual stimuli can also elicit negative associations following less rewarding sexual experiences, or if one generally has negative beliefs about sex. For instance, one might experience feelings of disgust when viewing or touching one’s own (or their partner’s) genitals [2], which may be acquired through conservative teachings that sex is ‘bad’, or through unpleasant sexual experiences, etc. [3,4]. Such negative associations may consequently elicit an aversion, rather than an appetite, toward certain sexual stimuli or situations. This, in turn, can hinder sexual functioning, especially when occurring in someone who would, in fact, wish to enjoy sex.

Hindered sexual functioning can sometimes be experienced as pain during sex, as is common in women who suffer from Genito-Pelvic/Pain Penetration Disorder (GPD). This condition, in which one experiences difficulty with vaginal penetration, genito-pelvic pain, and pelvic floor muscle contractions during penile-vaginal penetration (altogether resulting in fear of penetrative sex) [5], affects 14–34% of young women [6,7], and up to 45% of older women [6]. In these cases, negative emotions are commonly experienced during sex, which could, in turn, lead to decreased sexual arousal (and potentially decreased vaginal lubrication) and increased bodily tension (including the pelvic floor muscle region), altogether increasing the likelihood of penetration becoming painful, or even impossible. Furthermore, a vicious cycle might then more easily emerge, where the initial experience of pain during sex can form a memory expectation that the next time one has penetrative sex, they might experience pain again. This altogether can further weaken or inhibit sexual arousal, and the cycle can thereby continue [8–15].

Inhibited sexual arousal has accordingly been at the forefront for factors involved in painful sex, and within the last two decades, so too has heightened disgust [16]. Indeed, while sexual arousal facilitates sexual functioning and pleasure, the latter diminishes it [15]. These two emotions in particular have been shown in a multitude of studies not only to be elicited amongst sexually asymptomatic women observing sexual stimuli, but they have also been shown to negatively correlate with each other [17–21]. In other words, whilst both emotions can be experienced at the same time, the more dominant emotion may likely inhibit the other, which can consequently impact the direction of the behavioural response [22–24], and influence the pleasurability of the sexual interaction.

For instance, in an experimental study where women were allocated either to a sexual arousal, non-sexual positive arousal, or neutral control group where film clips were assigned to elicit the respective emotional state, it has been found that participants who were sexually aroused rated disgust-eliciting stimuli as less disgusting than the other groups, and even showed a higher propensity of physically interacting with these stimuli [18]. Similarly, an inverse relationship was shown in a conditioning study where participants viewed two pornographic films; one was paired with a disgust film, and the other was not paired with anything. The results showed that the film paired with disgust elicited less subjective sexual arousal (i.e., reporting *feeling* sexually aroused) than the latter film, and elicited lower genital arousal as well [19].

In sum, whether an appetitive or aversive emotional state is most salient can then influence whether pain may be reduced or heightened [25–30]. During sexual arousal and pleasure, for instance, endogenous opioids are released in the body, and are proposed to play a major role in pain reduction [31–34]. Conversely, in an unpleasant sexual interaction with heightened negative emotions, the pain-relieving effects otherwise experienced during sex may be hindered. On this basis, it had previously been hypothesized that low sexual arousal may generally enhance pain (including sexual pain), whereas high sexual arousal may generally heighten the threshold for experiencing pain [21,28,35].

Accordingly, previous experimental studies have demonstrated that sexual pleasure and orgasm can greatly reduce pain in women [36–39]. In one study, finger pain compression was better tolerated during masturbation among women [34]. This was also the case in a study utilizing self-imagery leading to orgasm – that is, without any masturbation [40]. In men, this pain-modulatory effect was even found during subjective sexual arousal during a cold pressor test – that is, self-reported sexual arousal not related (or leading) to orgasm [28]. However, this latter effect could not be replicated in women [41], which cast doubt on the validity of the hypothesis that high subjective sexual arousal would generally heighten the threshold for experiencing pain in women. Indeed, Lakhsassi et al. conducted two subsequent studies to assess whether subjective sexual arousal, elicited by a pornographic film stimulus, might reduce pain caused by a cold pressor test in women [21,35]. In the first study, heterosexual female participants were asked to view a pornographic film stimulus featuring a heterosexual couple, and rate their levels of sexual arousal and pain. Though the manipulation was successful in eliciting a significant level of sexual arousal, the results showed no significant effects of subjective sexual arousal on (cold pressor) pain [35]. To assess the possibility that feelings of disgust might have interfered with the results, a replication study was conducted where the same film was used, and participants were asked to rate their levels of sexual arousal as well as disgust. Evidently, this film stimulus did indeed elicit almost as much disgust as sexual arousal, emotions which are found to negatively correlate with each other [21]. It would therefore be reasonable to suspect that the failure of this previous study to find a pain-reducing effect of the exposure to sexually arousing porn might be explained by the concurrently-elicited high levels of disgust. To arrive at more firm conclusions, it would be critical to test the impact of porn clips that do not elicit a substantial level of disgust while eliciting sexual arousal in women.

Therefore, the current experimental study selected a porn clip which was shown in a previous study to be highly effective in eliciting sexual arousal in women, while simultaneously eliciting only minor levels of disgust [42–43]. We hypothesized that this new, more ‘female-friendly’, film would reduce pain elicited by a cold pressor test, and increase tolerance to the cold pressor in female participants – relative to the neutral control condition. Moreover, in order to further optimize the current manipulation and elicit sufficiently high levels of subjective sexual arousal (while minimizing the level of concurrently-elicited disgust), we made use of a fantasizing period for participants viewing the new porn clip. In short, the study was designed to test the hypothesis that when increased subjective sexual arousal is not paralleled by increased disgust, pain-reducing effects may be observed.

## Method

### Participants

We recruited 174 predominantly heterosexual, female, undergraduate participants in their 1st-year of psychology who were: aged 18 + , English-speaking, right-handed, and sexually asymptomatic (i.e., no sexual dysfunction inclusive of anorgasmia, inability to achieve sexual arousal, or vaginismus/ dyspareunia). Eligible participants must not: have participated in regular ice-bath training or winter swimming, have a strong dislike for pornography, or have any medical problems involving pain (e.g., chronic pain). Participants were randomly assigned to one of three conditions: SEX-O (i.e., Original Film) (*n* = 58), SEX-F (i.e., Female-friendly film) (*n* = 58), and NEUTRAL (*n* = 58). Exclusions from analyses were performed pre- or during the experiment if the standardized protocol was not met due to researcher or participant error. Although the G*Power analysis determined that a minimum sample size of 159 participants was needed for a statistical power of 0.8 and an effect size of 0.25 in a Fixed-Effects One-Way Analysis of Variance (ANOVA), we recruited additional participants to reach the goal of 174 participants in total and ensure sufficient power for analyses. All participants were granted student credits in compensation for their time. Data collection took place between November 25th, 2022, and March 2nd, 2023. The study was approved by the Ethics Committee of the Faculty of Behavioural and Social Sciences at University of Groningen (PSY-2122-S-0422 Porn and Pain) on November 22nd, 2022, and pre-registered on AsPredicted (#114098).

### Materials

#### Film Stimuli.

Three films were shown for each of the three conditions on a 90x52 cm TV screen, including: (i) a new erotic ‘female-friendly’ film to elicit higher levels of subjective sexual arousal with low levels of disgust, (ii) the original erotic film used in Lakhsassi et al.’s [21,35] studies (which was found to elicit similar levels of sexual arousal and disgust) to assess whether a difference exists with sexual stimuli that can elicit higher versus lower feelings of disgust, and (iii) a slightly adjusted colored train-ride film, similar to the dated black-and-white train-ride film used in Lakhsassi et al.’s [21,35] studies to elicit a neutral control state. All films had a maximum duration of six minutes.

The differences in content between the two porn clips were mainly related to the type of sexual activity; in the former, there was a shorter segment of oral sex and mutual masturbation prior to penile-vaginal penetration. The latter film, on the other hand, had a longer segment of progressive partner attraction, kissing, and undressing. Such features are generally more appealing to a female audience [44].

#### Tepid Water Bath.

A plastic container (39x28) filled with tepid water at a temperature ranging between 29–30 degrees Celsius (*M* = 29.50, *SD *= 0.24) was used to standardize all participants’ hands to a tepid baseline temperature prior to beginning the experiment. The temperature was regulated by pouring small amounts of boiling water from a kettle into the cold-water bath, and mixing thoroughly.

#### Cold Pressor Test (CPT).

A CPT was used as a pain stimulus in order to maintain the methodology used in previous studies assessing the influence of subjective sexual arousal on pain [i.e., 21, 28, 35, 41], as well as because it is an efficient stimulus with regard to simulating peripheral pain [45] and testing affective-motivational aspects of pain [30]. The set-up was the same as that in Lakhsassi et al.‘s study [21]; the same coolbox (26x39) was filled with water and cooled using 6–7 medium-sized Ziploc bags of ice cubes. The temperature was set between 1.9 and 2 degrees Celsius prior to each participant starting the experiment, and an aquarium pump circulated the water throughout the coolbox. Two thermometers attached to an internally-developed temperature device from Arduino hardware and software company were steadied on opposite ends of where the participant’s hand would be submerged in order to monitor the temperature. Temperature signals were measured via a physiological amplifier (Porti 7, from TMSi). A live-feed webcam facing the participant’s hand (above the CPT) was used for the researcher to see and record via an e-prime software programmed timer the moment at which the hand went into the water, and the moment it was removed.

### Procedure

Participants arrived to the lab and stated their booking ID number, confirmed that they were above the age of 18, and sanitized their hands for the purpose of maintaining hygiene when using the CPT. Next, they were invited to read the information and consent forms. In the meantime, the researcher made sure the tepid water bath and CPT were at the appropriate temperatures (that is, the CPT was at 1.88 for participants in SEX-O and NEUTRAL conditions, and 1.81 for those in the SEX-F condition, given that this latter condition involved a fantasy period. The tepid water was continuously checked prior to participant use). All temperatures were between 1.9 and 2 degrees Celsius by the time the participants began viewing their respective film.

**Fig 1:**
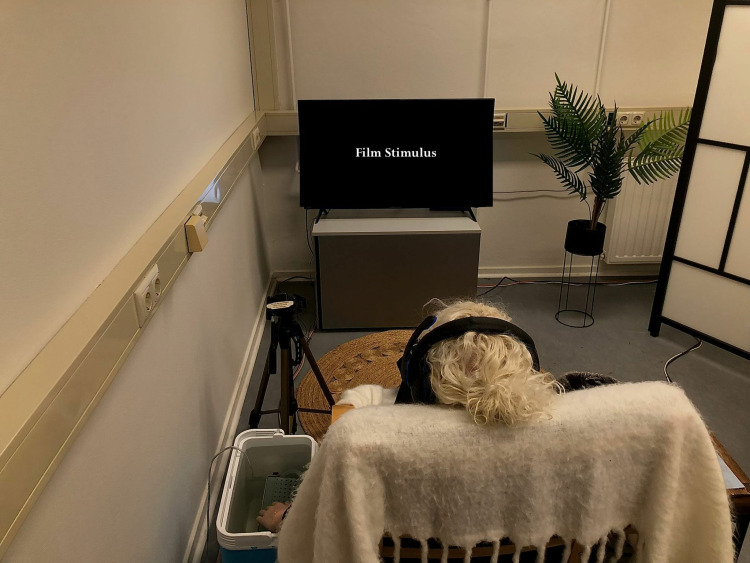
Experimental set-up. A research assistant was demonstrating the task. The image displays the TV screen with the film stimulus, the CPT on the left-hand side, the webcam tripod hovering over the CPT, headphones to listen to the film, and a cozy lab environment. A mouse was placed on the right-hand side table for the participants to answer the questions on-screen.

Once participants had signed the forms and given informed consent in writing, they were asked to remove their watch and jewelry (if applicable) in order to prepare for the CPT. For transparency purposes, they were shown the CPT live-feed camera installed for timing the CPT duration, and were informed that nothing would be recorded. Afterwards, participants were asked to place their left hand (non-dominant hand) in the tepid water bath for 1 minute. The non-dominant hand was selected because tolerance is found to be significantly longer in the dominant hand [46]. Once they dried their hands, they were asked to sit facing the TV screen (see Fig 1 for experimental set-up), and the experimental procedure was explained in detail.

In order to enhance feelings of sexual arousal without concurrently eliciting disgust, the SEX-F condition started with a 2-min period of fantasizing. Here, participants were prompted to close their eyes and think about a past enjoyable and pleasurable sexual experience, and allow themselves to fantasize about it. On the chance that they had no such memory, they were asked to think of a sexual fantasy that they would enjoy, were it to occur. After 2-minutes, a light bell would sound, signaling that they could open their eyes. They were subsequently asked to confirm whether they managed to follow the exercise by ticking the on-screen ‘yes’ or ‘no’ buttons. However, due to programming error, the responses could not be seen.

Next, the screen stated that a film clip would begin to play, and instructed participants to focus their attention on the film and follow the prompts on the screen as they appeared. Once ready, they could press ‘start’, and the assigned film would begin. After 1 minute and 41 seconds, a VAS (visual analogue scale where participants could not see any scale numbers) appeared below the film, asking “*how sexually aroused do you feel?*” from ‘*not at all’* on the far left, to ‘*very strongly’* on the far right of the scale. There was an 8-second maximum timespan to respond before the question moved on to the next VAS, which stated “*how disgusted do you feel?*” for another 8-second maximum duration. As soon as the questions were answered, the scale would vanish. Two minutes after the start of the film, the screen would show an instruction underneath the film, stating: *Place your hand in the water and leave it in for as long as you can tolerate.* Both VAS questions and the CPT instruction were displayed while the film continued playing. Once the participants removed their hand, or, once the 4-minute maximum CPT duration was reached, the timer and film would stop, and the screen would instruct participants to answer the remaining questions and dry their hands with the paper towel on the side table. The VAS questions stated “*How intense was the pain you felt?*”, “*How sexually aroused did you feel during the experiment?*”, and “*How disgusted did you feel during the experiment?*”.

### Data Analyses

Four one-way ANOVAs with Condition as a 3-level factor (one for each VAS relating to pre and post CPT ratings of sexual arousal and disgust) were used to assess whether the experimental manipulations had successfully elicited the target emotional states; that is, whether the SEX-F condition elicit substantially more sexual arousal and less disgust than the latter two conditions. Next, we checked ANOVA assumptions prior to hypothesis testing using Q-Q plots and histograms to assess normality of residuals, and using the Levene’s test to assess homoscedasticity. Subsequently, two one-way between-group ANOVAs were used to assess subjective pain ratings and CPT duration across conditions. These same ANOVAs were conducted again after removing outliers based on the 1.5 x interquartile range rule. Finally, to examine whether feelings of sexual arousal and disgust negatively correlate, as suggested by previous studies, we conducted correlational analyses between these two emotions in each SEX condition. All statistical analyses were performed on SPSS Statistics (version 28).

## Results

### Assumptions

A histogram and Q-Q plot analysis for CPT duration showed that the unstandardized residuals were positively skewed, while those for subjective pain ratings were negatively skewed. Nonetheless, we performed our analyses as planned given that ANOVAs remain robust against deviations from normality [47]. The homogeneity of variances assumption was based on the median, given our asymmetric distributions [48]. The assumption was neither violated for CPT duration (Levene’s statistic (2, 171) = 2.63, *p* = .08), nor for subjective pain ratings (Levene’s statistic (2, 171) = 0.44, *p* = .65).

### Manipulation Checks

Data was missing in both pre-CPT VASs (*n* = 4 for the sexual arousal VAS; *n* = 6 for the disgust VAS). Little’s MCAR test showed that the data was missing at random (Chi-square = 1.29, *df* = 2, *p* = .53). Thus, we used the expectation maximization technique to estimate the missing values prior to the CPT.

#### Emotional State Induction Assessed Prior to the CPT.

A one-way ANOVA assessing the sexual arousal manipulation across conditions (*F* (2, 171) = 90.90, *p* < .001) showed that the SEX-F condition had the highest sexual arousal rating (*M* = 45.86, *SD* = 19.19), although it did not significantly differ from that of the SEX-O condition (*M* = 39.72, *SD* = 22.53, *p* = .15) according to a Tukey HSD post hoc test. Both SEX conditions significantly differed in sexual arousal from the NEUTRAL condition (*M* = 4.59, *SD* = 8.56, *ps* < .001). A one-way ANOVA assessing the disgust manipulation across conditions (*F* (2, 171) = 43.07, *p* < .001) subsequently showed that the SEX-O condition had the highest disgust rating (*M* = 35.07, *SD* = 24.40), which significantly differed from the SEX-F condition (*M* = 23.17, *SD* = 18.27, *p* = .002), according to a Tukey HSD post hoc test. Both SEX conditions also significantly differed from the NEUTRAL condition (*M* = 3.60, *SD* = 9.56, *ps* < .001).

#### Emotional State Induction Assessed After the CPT.

A subsequent one-way ANOVA assessing the sexual arousal manipulation after the CPT across conditions (*F* (2, 171) = 82.94, *p* < .001) showed that the SEX-F condition had the highest sexual arousal rating (*M* = 45.79, *SD* = 21.04), which significantly differed from both the SEX-O condition (*M* = 36.19, *SD* = 25.49, *p* = .02) and the NEUTRAL condition (*M* = 2.07, *SD* = 3.94, *p* < .001), according to a Tukey HSD post hoc test. A one-way ANOVA assessing the disgust manipulation after the CPT across conditions (*F* (2, 171) = 31.67, *p *< .001) subsequently showed that the SEX-O condition had the highest disgust rating (*M* = 34.02, *SD* = 28.70), followed by the SEX-F condition (*M* = 24.47, *SD* = 23.36, *p* = .05), followed by the NEUTRAL condition (*M* = 2.43, *SD* = 8.51, *p* < .001), according to a Tukey HSD post hoc test. Both SEX conditions significantly differed from the NEUTRAL condition.

### Hypothesis Testing

**Fig 2:**
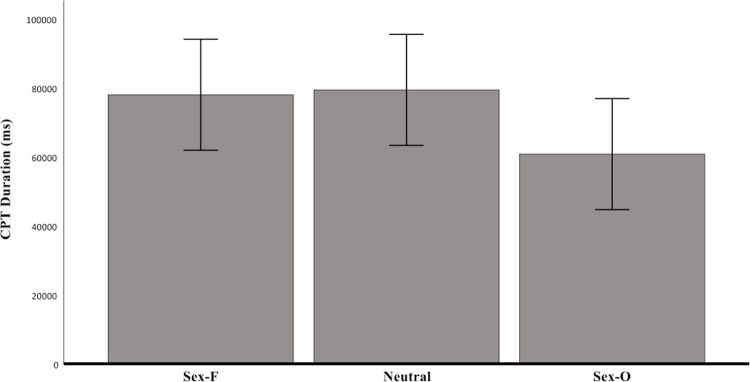
CPT duration in milliseconds as a function of condition. No significant differences in CPT duration were observed, although a non-significant mean difference of nearly 20 seconds could be observed between the SEX-O and SEX-F conditions.

**Fig 3:**
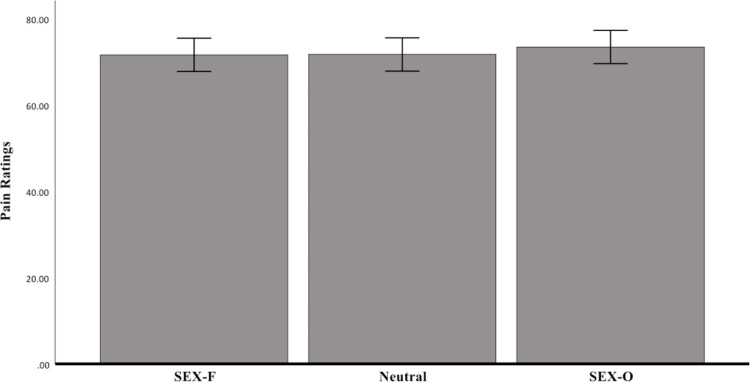
Self-reported pain ratings as a function of condition. No significant differences in pain ratings were observed.

Two one-way ANOVAs showed that there were no significant differences across conditions in CPT duration (*F* (2, 171) = 1.62, *p *= .20, *η*_*p*_^*2*^ = 0.02) and subjective pain ratings (*F* (2, 171) = 0.27, *p *= .76, *η*_*p*_^*2*^ = 0.003) (see Figs 2–3 for an illustration of the mean scores). After having removed the 1.5x interquartile range outliers, no significant differences were found across conditions either.

### Exploratory Analyses

**Fig 4:**
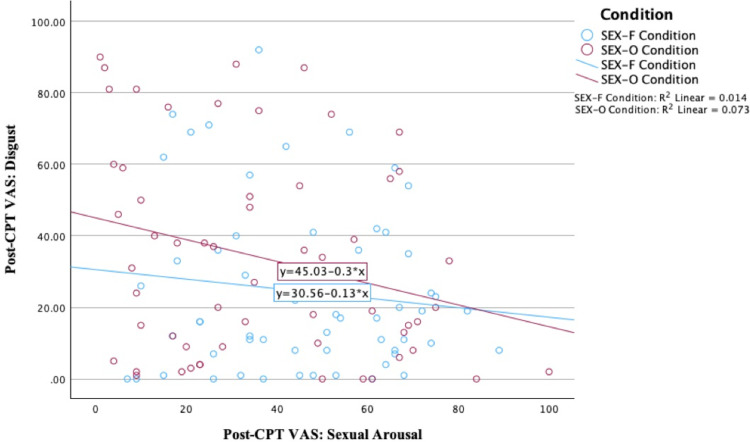
Mean correlations between sexual arousal and disgust, measured after the CPT in both SEX conditions. A significant effect is shown between sexual arousal and disgust in the SEX-O condition, and a similar non-significant pattern is illustrated in the SEX-F condition.

Bivariate correlations were computed in the SEX-F condition with sexual arousal and disgust in both pairs of pre and post-CPT VASs. Unexpectedly, no significant correlations were found. In the SEX-O condition, we found a significant small negative correlation between the post-CPT VASs assessing sexual arousal and disgust (*r* = -0.27, *p* = 0.04). These latter results replicate those from previous research using the same SEX-O film. See Fig 4 for a visual illustration of the correlations.

## Discussion

The present study is the third in a triad of studies testing whether subjective sexual arousal has the capacity to reduce experimentally-elicited cold pressor pain in young, female participants with no sexual dysfunctions. Here, we examined whether a porn film with high sexual arousal ratings and relatively low concurrent disgust ratings might reduce pain and increase tolerance to pain, in comparison to the previous porn film used in the former two studies, and in comparison to a neutral condition. Despite the new film (SEX-F) having been found to be significantly more sexually arousing than its former counterpart (SEX-O), as well as less disgusting, this nonetheless resulted neither in pain reduction, nor in increased pain tolerance.

Moreover, sexual arousal and disgust ratings were paradoxically not shown to significantly correlate in the SEX-F condition, whereas in the SEX-O condition, a significant negative correlation was found, thereby replicating previous findings using the same film. The film with the largest difference between sexual arousal and disgust ratings (i.e., SEX-F) was the one where the two emotions did not significantly correlate. Perhaps this points to the possibility that these emotions might not correlate when they are not experienced to the same, or similar degree, as they had been with the SEX-O film. Specifically, the SEX-F film might be taken as less ambiguous in its sexually arousing properties, under which condition a relatively minor level of concurrently-elicited disgust might not detract from the arousing properties. On the other hand, the SEX-O film might elicit more ambivalence in participants. In this case, for some participants, the disgusting features might dominate, thereby reducing the impact of the arousing properties of the film, whereas for others, the arousing features might dominate, thereby reducing the impact of the disgust-eliciting properties. Thus, while research findings are mixed with regard to finding a negative correlation between these emotions (and the underlying reasons are not yet clear) [see, e.g., 49–51], such differences in sexual arousal and disgust elicited in each film might perhaps offer one explanation for why these emotions sometimes do not correlate.

Another consideration is that the ways in which these two emotions correspond with each other might be dependent upon individual features. For instance, in a previous study, it has been found that in women with high disgust sensitivity, sexual arousal was reduced when they were exposed to sexual body fluids, whereas in women with low disgust sensitivity, sexual arousal was increased [49]. Future studies could therefore differentiate between participants who are high or low in disgust sensitivity when eliciting sexual arousal. Similarly, future studies could benefit from also differentiating between participants with high sexual excitations from those with high sexual inhibition using the sexual inhibition/sexual excitation scales [52].

Despite all methodological amendments applied to the current study for the purpose of optimizing the design and reducing potential confounding variables from previous studies (e.g., reducing disgust, heightening sexual arousal, utilizing film stimuli instead of photo stimuli to elicit sexual arousal, and assessing pain tolerance as well as pain intensity) [see 35], the findings provided no support for the view that subjective sexual arousal generally reduces pain, or increases pain tolerance. The current findings thus add to the view that, in women, subjective sexual arousal does not generally increase the threshold for experiencing pain, nor does it reduce pain intensity. Rather, this effect seems to require self-stimulation and/or orgasm [see, e.g., 34, 40]. In men, on the other hand, subjective sexual arousal seems sufficient [28]. However, since neither the study conducted on men, nor this current study, had utilized any genital measures of sexual arousal, it remains possible that for men, it was in fact objective (physiological) sexual arousal that modulated their pain. Indeed, studies have found that men tend to have a much higher concordance rate between genital arousal and self-reported sexual arousal in comparison to women [53–54]. Thus, perhaps women in these studies were not as genitally aroused as men were, and reaching a particular level of genital arousal may be a requirement without which pain modulation might not be reached.

Alternatively, another potential explanation could be related to a possible sex difference in the release of opioids, which are highly potent natural analgesics typically released during sex (as well as other activities) [32–34]. For instance, opioids are proposed to be present in low doses during sexual arousal prior to sexual activity [55], as indicated by increased motivation in male rats to chase after a sexually receptive female rat following an endorphin injection [31]. However, animal studies have also found that following morphine injections, male rats experienced significantly greater analgesic effects than female rats [56–57]. Similar sex differences were found in human studies showing a weaker opioid-related analgesic response in women compared to men [58]. Perhaps such differences in the opioid system might possibly account for the apparent sex differences in pain modulation in response to heightened subjective sexual arousal in participants.

### Limitations

It is worth noting that pain sensitivities may differ across the body, implying that cold pressor pain may respond differently to sexual arousal than genital pain. Thus, future research might also consider replicating this study with vulvovaginal pain instead of cold pressor pain in order to more closely reflect the type of pain experiences in women with GPD. For instance, future studies might consider utilizing a vaginal pressure inducer, a device designed to mimic the vaginal penetration [59], which might elicit a type of pain in some participants that is more similar to the pain experienced in women with GPD. Furthermore, despite all efforts to minimize feelings of disgust, the SEX-F porn stimulus still elicited some disgust. This effect seems inherent to viewing a porn film for women, and may perhaps be unavoidable. Future studies might therefore consider utilizing different methods of inducing subjective sexual arousal (e.g., fantasy) in order to further minimize disgust or other negative emotions.

Furthermore, we focused on female participants without sexual disorders with the idea that the findings could provide clues as to how subjective sexual arousal might generally influence pain, including genital pain as typically reported by women with GPD. If indeed, heightened subjective sexual arousal would have generally reduced pain in women, this would have suggested that experiencing high subjective sexual arousal prior to potentially painful sexual activity (i.e., sexual penetration) might help prevent the development of (chronic) sexual pain, as experienced in women with GPD. However, since the current study provided no evidence to support the hypothesis that subjective sexual arousal generally reduces pain, the current results cast doubt on the view that low subjective sexual arousal might explain genital pain in women with GPD. Yet, as already mentioned above, it remains to be tested if perhaps, specifically within the context of genital pain (instead of cold-pressor pain), sexual arousal does attenuate the experience of pain. If this would indeed be the case, it would be relevant to test if such effects would also be evident in women with GPD. It would additionally be helpful in such a study to assess concurrent negative emotions (beyond disgust), given that women with GPD in particular are usually even more susceptible to negative emotions during sexual activity than women with no sexual pain complaints [4,60,61].

Finally, it is noteworthy that while participants were viewing their respective sex stimulus prior to the CPT instruction, the SEX-F film was not significantly more arousing than the SEX-O film, as originally expected. However, the sexual arousal levels had changed post CPT, as the SEX-O film gradually decreased in sexual arousal ratings and significantly differed from the SEX-F film by the end of the experiment. Importantly, the SEX-O film maintained significantly higher disgust ratings than then SEX-F film pre-CPT, and near traditional significance levels (*p* = 0.052) post-CPT. Thus, average disgust levels remained somewhat stable throughout, and highest within the SEX-O film, as expected. Nonetheless, it would have ultimately been further informative if the SEX-F film were consistently more sexually arousing and less disgust-eliciting than the SEX-O film from the start of the experiment to the end, and not only in retrospect at the end. Previous studies have attempted to accomplish this through minimizing potential confounds, for instance, utilizing sex stimuli that did not contain depictions of coitus in order to avoid provoking a threat response from the stimulus itself in some participants [10]. Future studies may also consider screening their sample for other potential confounds (e.g., being sexually inactive, or having any pre-existing aversions toward sexual activity, sexual excitation/inhibition propensities, anxiety, depression, porn consumption, etc.). Despite utilizing a randomized experimental design, there is always a chance of uneven distribution, and such screening could help to post-hoc ascertain whether this might have taken place.

## Conclusion

In line with previous studies, the current findings provided no support for the idea that subjective sexual arousal generally reduces the experience of acute pain in healthy women. This seems to be the case despite efforts to minimize feelings of disgust in response to sexual stimuli. However, as this study relied on a generic model of pain, it cannot be ruled out that a different pattern would emerge when specifically targeting genital pain. It thus remains to be tested whether the current findings can be generalized to genital pain.
